# The cytochrome P450 Cyp6t3 is not required for ecdysone biosynthesis in *Drosophila melanogaster*

**DOI:** 10.17912/micropub.biology.000611

**Published:** 2022-08-03

**Authors:** MaryJane Shimell, Michael B O'Connor

**Affiliations:** 1 University of Minnesota

## Abstract

The steroid hormone 20-hydroxyecdysone (20E) is essential for proper development and the timing of intermediary stage transitions in insects. As a result, there is intense interest in identifying and defining the roles of the enzymes and signaling pathways that regulate 20E production in the prothoracic gland (PG), the major endocrine organ of juvenile insect phases. Transcriptomics is one powerful tool that has been used to identify novel genes that are up- or down-regulated in the PG which may contribute to 20E regulation. Additional functional characterization of putative regulatory candidate genes typically involves qRT-PCR and/or RNAi mediated knockdown of the candidate mRNA in the PG to assess whether the gene’s expression shows temporal regulation in the PG and whether its expression is essential for proper 20E production and the correct timing of developmental transitions. While these methods have proved fruitful for identifying novel regulators of 20E production, characterizing the null phenotype of putative regulatory genes is the gold standard for assigning gene function since RNAi is known to generate various types of “off target” effects. Here we describe the genetic null mutant phenotype of the *Drosophila melanogaster* *Cyp6t3* gene *. Cyp6t3* was originally identified as a differentially regulated gene in a PG microarray screen and assigned a place in the “Black Box” step of the E biosynthetic pathway based on RNAi mediated knockdown phenotypes and rescue experiments involving feeding of various intermediate compounds of the E biosynthetic pathway. In contrast, we find that Crispr generated null mutations in *Cyp6t3* are viable and have normal developmental timing. Therefore, we conclude that Cyp6t3 is not required for E production under typical lab growth conditions and therefore is not an obligate enzymatic component of the Black Box.

**Figure 1.**
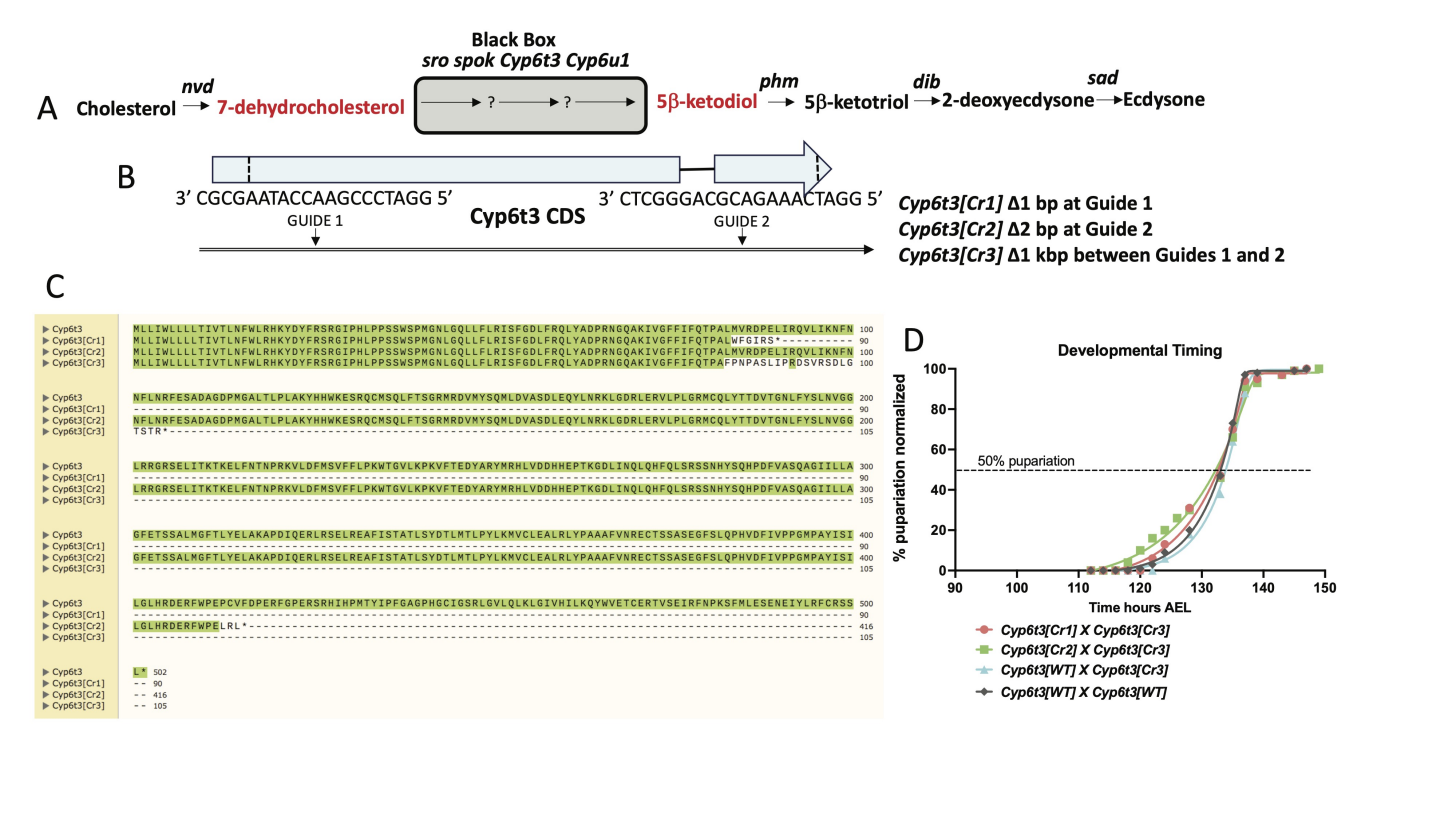
**A.** **Ecdysone biosynthetic pathway.** The feeding substrates for placement in the Black Box are shown in red. Number of arrows in the Black Box is for illustrative purpose only. **B. Cartoon of the *Cyp6t3* genomic region.** Exons are shown in light blue and dashed lines denote the Cytochrome P450 domain. Sequences for the guide RNAs are shown in anti-sense because the targeted sequence is the negative DNA strand. Three new alleles are described below. **C. Clustal alignment** . Cyp6t3 aligned with Cyp6t3 mutant proteins. Pale green highlight indicates amino acid identity. Note that Cyp6t3[Cr1] and Cyp6t3[Cr3] are early truncations, while more than 80% of the protein is retained in Cyp6t3[Cr2]. **D. Developmental timing.** *Cyp6t3* transheterozygous combinations and wild type (black line). Non-linear regression using Prism software with 50% pupariation indicated by dashed line.

## Description

In all Arthropods, the steroid hormone ecdysone (E) acts to control molting and metamorphosis (Qu et al. 2015). During the past two decades, many of the enzymatic steps that convert dietary cholesterol to ecdysone in the insect *Drosophila melanogaster* have been identified and their activities ordered within a biosynthetic pathway. (Figure 1A, Chávez et al. 2000, Warren et al. 2002, Warren et al. 2004, Niwa et al. 2004, Yoshiyama-Yanagawa et al. 2011). Collectively, these genes are referred to as the Halloween group (Warren et al. 2002) and include *phantom (phm), disembodied (dib), shadow (sad), shroud (sro),* and *spook (spo)* which were first identified in the embryonic lethal screens of Nüsslein-Volhard, Wieschaus, and Jürgens as mutants that failed to produce differentiated cuticle (Nüsslein-Volhard et al. 1984, Wieschaus et al. 1984, Jürgens et al. 1984). A closer examination of the embryo proper revealed that null mutants in these genes all exhibit a common phenotype characterized by arrest at embryonic stages 15-17 with failures of head involution, dorsal closure, and cuticle production (Chávez et al. 2000, Warren et al. 2002, Petryk et al. 2003, Niwa et al. 2004, Warren et al. 2004). Subsequent genetic and biochemical analysis demonstrated that each of these gene products acts at a specific step in the biosynthetic pathway and their loss results in failure to synthesize ecdysone, and hence they all produce an identical loss-of-function phenotype.

Despite this progress, there remains uncertainty in the enzymes and reactions involved in an early step of the pathway termed the “Black Box”. The name arises because the chemical intermediates between the upstream 7-dehydrocholesterol (7dC) and the downstream 5beta-ketodiol have eluded isolation (Gilbert et al. 2002). Nevertheless, the activity of several gene products including Shroud (Sro), Spo/Spok, Cyp6t3 and Cyp6u1 have been suggested to act within the Black Box based on rescue experiments that involve feeding mutant larvae intermediate compounds that are thought to be upstream and downstream of the Black Box enzymes. In these experiments, feeding the downstream intermediate 5beta-ketodiol rescues loss-of-function larvae and enables them to progress through additional molts while feeding the upstream intermediate 7dC does not (Figure 1A). (Niwa et al. 2010; Ono et al. 2006 *;* Ou et al. 2011; Christesen et al. 2017).

Since all Halloween null mutants are embryonic lethal, the feeding experiments which helped position various gene products in the biosynthetic pathway sometimes involved bypassing the embryonic lethality by using RNAi to specifically knockdown the function of these genes in the prothoracic gland (PG), the source of larval ecdysone. The embryonic tissue source of E is still unknown. Strong RNAi knockdown in the PG of any of the Halloween gene products leads to first instar arrest. However, feeding the larvae various intermediates often enables them to progress into the second and third instar stage, provided the intermediate is downstream of the enzymatic block. These types of experiments suggested that the Sro and Spo/Spok enzymes are components of the Black Box.

The identification of *Cyp6t3* (FBgn0033697) and *Cyp6u1* (FBgn0033121) genes as encoding possible factors of the E biosynthetic pathway came about differently from classical genetic studies. *Cyp6t3* was identified as a gene that was specifically up-regulated in the PG in response to loss of the transcription factor DHR4. (Ou et al. 2011). *DHR4* mutants pupariate early likely because of precocious ecdysone production, and simultaneous knockdown of *Cyp6t3* in a *DHR4* mutant background suppressed the early pupation phenotype. In addition, RNAi knockdown of *Cyp6t3* in the PGs of wild type animals resulted in slow growth and large pupae, both of which were suppressed by feeding knockdown larvae ecdysone or 5beta-ketodiol but not 7-dC suggesting that, like Spo/Spok and Sro, this enzyme works at the Black Box step. Because of the low transcript abundance of *Cyp6t3* , it was also hypothesized that *Cyp6t3* might be a “bottleneck” in ecdysone biosynthesis, or even rate limiting. *Cyp6u1* was identified in a genome wide transcriptome analysis of the PG as a P450 that showed high level expression specifically in the PG, and RNAi knockdown in the PG led to a developmental delay/arrest phenotype which was rescued by feeding ecdysone or 5beta-ketodiol but not 7-dC, again positioning this enzyme at the Black Box step.

Since it has been reported that tissue-specific knockdown of genes can have more severe phenotypes than genetic null mutations (Gibbens et al. 2011, Ohhara et al. 2015), we made Crispr-mediated null mutations in *Cyp6t3* as an additional means to assess its function during *Drosophila* development *.* Guide RNAs from both ends of the *Cyp6t3* cytochrome P450 domain were simultaneously injected into a Cas9 stock (Bestgene Inc.) for the purpose of generating mutations at each individual site as well as potential deletions between the sites. After screening the progeny of the G0 flies, we obtained 3 mutations *Cyp6t3[Cr1]* , a 1 bp deletion at Guide 1, *Cyp6t3[Cr2]* , a 2 bp deletion at Guide 2, and *Cyp6t3[Cr3]* , a 1 kbp deletion between Guides 1 and 2 (Figure 1B). Translation of these mutated genes yields proteins with different premature stops or a large deletion within the Cytochrome P450 domain of Cyp6t3 (Figure 1C) *.* All three mutations are homozygous viable. For this reason alone, we deemed *Cyp6t3* a non-essential gene for ecdysone biosynthesis.

Although loss of *Cyp6t3* is not essential for animal survival, we reasoned that it could affect the rate of ecdysone biosynthesis. Previous work has shown that either raising or lowering the basal E biosynthetic rate leads to advanced or delayed timing of pupariation, respectively (Ou et al. 2016, Koyama et al. 2014, and Shimell et al. 2018). To address this issue, we assessed the time to pupariation of various *Cyp6t3* mutant combinations (Figure 1D). In sum, we find no delay in developmental timing compared to the wild type control suggesting that the basal rate of ecdysone synthesis in the *Cyp6t3* mutants is on par with wild type.

The fact that *Cyp6t3* is not an essential component of the E biosynthetic pathway under lab growth conditions should not be surprising since, unlike the other Halloween genes that are highly conserved across all arthropods examined to date, *Cyp6t3* has been lost in some clades (Ou et al. 2011 and Good RT et al. 2014). In addition, as previously noted, the transcript abundance of *Cypt6t3* is very low by qRT-PCR (Ou et al. 2011) and did not make the cutoff in 2 different ring gland-specific transcriptional profiles, the only putative E biosynthetic P450 gene failing to do so (Ou et al. 2016 and Christesen et al. 2017). Despite these observations, the regulation of *Cyp6t3* transcription by DHR4 and its ability to suppress the precocious *DHR4* mutant phenotype when knocked down in the PG remains intriguing. It would be interesting to determine whether a *Cyp6t3,* *DHR[1]* double mutant shows the same suppression of precocious pupariation as found when RNAi is used to knockdown *Cyp6t3* in the PG of a *DHR[1]* mutant. It is also possible that Cyp6t3 is important under specialized growth conditions that modulate DHR4 activity. Another possibility is that Cyp6t3 function is redundant in *D. melanogaster* , and perhaps other species, with another P450 enzyme. Precedence for the existence of partial redundance in E biosynthetic enzymes in insects is exemplified by the Spo/Spok relationship (Ono et al. 2006). Both are thought to function within the Black Box but at different developmental stages, Spo acting embryonically and in female follicle cells while Spok acts in the PG during larval stages. One intriguing candidate for a redundant activity could be *Cyp6u1* . This gene product is also a member of the P450 clade 6 subgroup but is highly conserved across Arthropoda (Good RT et al. 2014). Examining the phenotype of *Cyp6u1* null mutants and *Cyp6t3;Cyp6u1* double mutants might provide insight on the redundancy issue.

In summary, our studies do not resolve the enigma of the Black Box reaction mechanism in the E biosynthetic pathway. However, our elimination of Cyp6t3 involvement under standard growth conditions helps reduce the complexity of the necessary components. This should aid in simplifying the design of future studies aimed at understanding the details of the biochemical reactions catalyzed by the Blackbox enzymes and whether their regulation serves as a rate limiting step in E biosynthesis as previously suggested (Gilbert et al. 2002).

## Methods

Crispr mutant generation

The CRISPR Optimal Target Finder (Gratz et al. 2014) was employed to identify two target sites within *Cyp6t3* with no off-target sites. The targets with the PAM are: 5’ GGATCCCG¯AACCATAAGCGC CGG 3’ and 5’ GGATCAAA ¯ GACGCAGGGCTC CGG 3’. Sense and antisense oligonucleotides (Oligo Table) for the two guides RNAs were cloned into pU6-BbsI-chiRNA (Addgene #45946; Gratz et al. 2013). Both guide RNAs were injected into vas-Cas9 embryos (BDSC51323, BestGene, Chino Hills CA). Single G0 flies were screened by PCR using primers *cyp6t3 01F* and *cyp6t3 02R* (Oligo Table) for the presence of deletions, making them candidates for further analysis. Ten to fifteen individual F1 progeny of G0 flies that displayed multiple deletion bands by PCR (possible Cr mutant/+) were then crossed to a CyO* balancer (BDSC 3198), homozygosed, the DNA region amplified by PCR and then sequenced for the presence of a mutation and its precise location. Both *Cyp6t3[Cr1]* and *Cyp6t3[Cr2]* mutations were obtained from a single G0 fly.

Clustal omega analysis used SnapGene Version 6.0.7 software.

Developmental timing

Crosses were set up in cages. Four-hour collections of embryos on yeast-apple juice plates (yeast paste spread on 25% apple juice, 25 mg/mL dextrose, 1.5 mg/mL tegosept, 2.2% agar) were aged about 16 hours, until 1 ^st^ instar larvae started to emerge. 30-35 larvae were transferred to vial food (Bloomington standard cornmeal food) sprinkled with dry yeast and allowed to grow at 25° with a 12-hour light/dark cycle. Pupariation was scored every 2 hours with the data from 4 vials combined due to the speed of pupariation and lack of data points necessary for non-linear regression analysis in Prism (asymmetric Sigmoidal, 5PL).

## Reagents

Oligo Table

**Table:** 

CTTCGGATCCCGAACCATAAGCGC	sense Guide 1	Crispr
AAACGCGCTTATGGTTCGGGATCC	antisense Guide 1	Crispr
CTTCGGATCAAAGACGCAGGGCTC	sense Guide 2	Crispr
AAACGAGCCCTGCGTCTTTGATCC	antisense Guide 2	Crispr
GTCCCATCAGGTTGCCC	*cyp6t3 01F*	PCR, sequencing
GCAGAGCTTTGAATCGAATTTGG	*cyp6t3 02R*	PCR, sequencing

Fly lines

**Table:** 

*y[1] M{RFP[3xP3.PB] GFP[E.3xP3]=vas-Cas9}ZH-2A w[1118]/FM7c*	BDSC 51323
w[*]; sna[Sco]/CyO, S[*] bw[1]	BDSC 3198
